# Cardiac Sarcoidosis Culminating in Severe Biventricular Failure

**DOI:** 10.1155/2009/856785

**Published:** 2009-09-06

**Authors:** Takefumi Ozaki, Noritomo Ohnuma, Norihiro Shimizu, Atsushi Hasegawa, Masashi Horimoto

**Affiliations:** ^1^Division of Cardiovascular Disease, Chitose City Hospital, Chitose City, 066-8550, Japan; ^2^Division of Internal Medicine, Chitose City Hospital, Chitose City, 066-8550, Japan

## Abstract

A 59-year-old woman with a history of lung sarcoidosis developed general edema and exertional dyspnea. An electrocardiogram showed first-degree atrioventricular block with complete right bundle branch block. Chest X-ray showed cardiomegaly. Echocardiography showed diffuse and severe hypokinesis of the left ventricle (LV) and biventricular enlargement with severe tricuspid regurgitation. Myocardial scintigraphy disclosed a perfusion defect at the ventricular septum and hypoperfusion at the posterior wall and the apex. On cardiac catheterization, pulmonary capillary wedge pressure, right ventricular, and right atrial pressures were elevated. Coronary angiograms were normal. Myocardial biopsy of the right ventricle histologically revealed epithelioid cell granuloma with infiltration of fibrous cells. The patient's symptom and LV function were improved with conventional medical therapy for heart failure. This is a rare case of cardiac sarcoidosis resulting in biventricular failure.

## 1. Introduction

Sarcoidosis is a granulomatous disease of unknown etiology that primarily affects the lung and infrequently myocardium. Myocardial sarcoidosis is known to cause cardiac arrhythmias, electrical conduction disturbance, and rarely congestive heart failure [1]. In cardiac sarcoidosis, left ventricle (LV) is more frequently affected than the right ventricle (RV) and biventricular failure is rare. We report a case of cardiac sarcoidosis complicated by severe biventricular failure. The possibility of RV involvement by cardiac sarcoidosis was also discussed.

## 2. Case Presentation

A 59-year-old woman was admitted to the hospital in January 2001 due to exertional dyspnea and general edema with a functional class III of New York Heart Association (NYHA), which started from October 2000. She had suffered lung sarcoidosis in 1987, when bilateral hilar lymphadenopathy was noted on chest X-ray. She received no medication due to absence of symptoms and organ dysfunctions.

**Figure 1:**
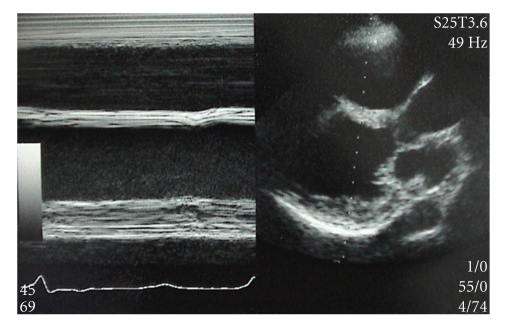
Echocardiography shows biventricular enlargement and diffuse hypokinesis of the left ventricle without wall thinning.

On physical examination, blood pressure was 142/90 mm Hg and pulse was regular with 120 bpm. Pretibial and facial edema was noted without swelling of superficial lymph nodes. Heart and respiratory sounds were normal. Chest X-ray showed cardiomegaly with a cardiothoracic ratio of 0.67 but normal lung field. Computed tomography of the chest and ultrasound of the abdomen disclosed enlarged lymph nodes at the mediastinum and the hepatic hilus, respectively. An electrocardiogram (ECG) showed sinus tachycardia and first-degree atrioventricular block with complete right-bundle-branch-block. A Holter ECG monitoring during 24 hours showed 1275 beats of ventricular extrasystole with Lown grade 4B and 21 beats of supraventricular extrasystole. Two-dimensional echocardiography showed enlarged biventricles; left ventricular (LV) and right ventricular (RV) end-diastolic dimension were 58 mm and 45 mm, respectively (Figure 1). Diffuse and severe hypokinesis of the LV with ejection fraction (EF) of 25% and fractional shortening (FS) of 12% was shown without thinning of the LV wall. Color Doppler echocardiography showed severe tricuspid regurgitation.

On laboratory data, complete blood count and C-reactive protein level were normal. Liver, renal, and thyroid functions were also normal. Serum concentrations of lysozyme and brain natriuretic peptide were increased to 12.9 *μ*g/mL and 500.9 pg/mL, respectively, while the concentration of angiotension-converting enzyme was normal. Right heart catheterization revealed elevations of pulmonary capillary wedge pressure (mean 30 mm Hg), pulmonary artery pressure (systolic 37 mm Hg, diastolic 30 mm Hg, mean 33 mm Hg), RV ventricular pressure (systolic 36 mm Hg, diastolic 20 mm Hg, end-diastolic 22 mm Hg), and right atrial pressure (mean 23 mm Hg). Coronary angiograms were normal but left ventriculography showed a diffuse reduction in LV motion, especially in the anteroseptal and apical wall motion. A His-bundle electrocardiogram showed prolonged AH (164 msec) and HV (60 msec) intervals.

**Figure 2:**
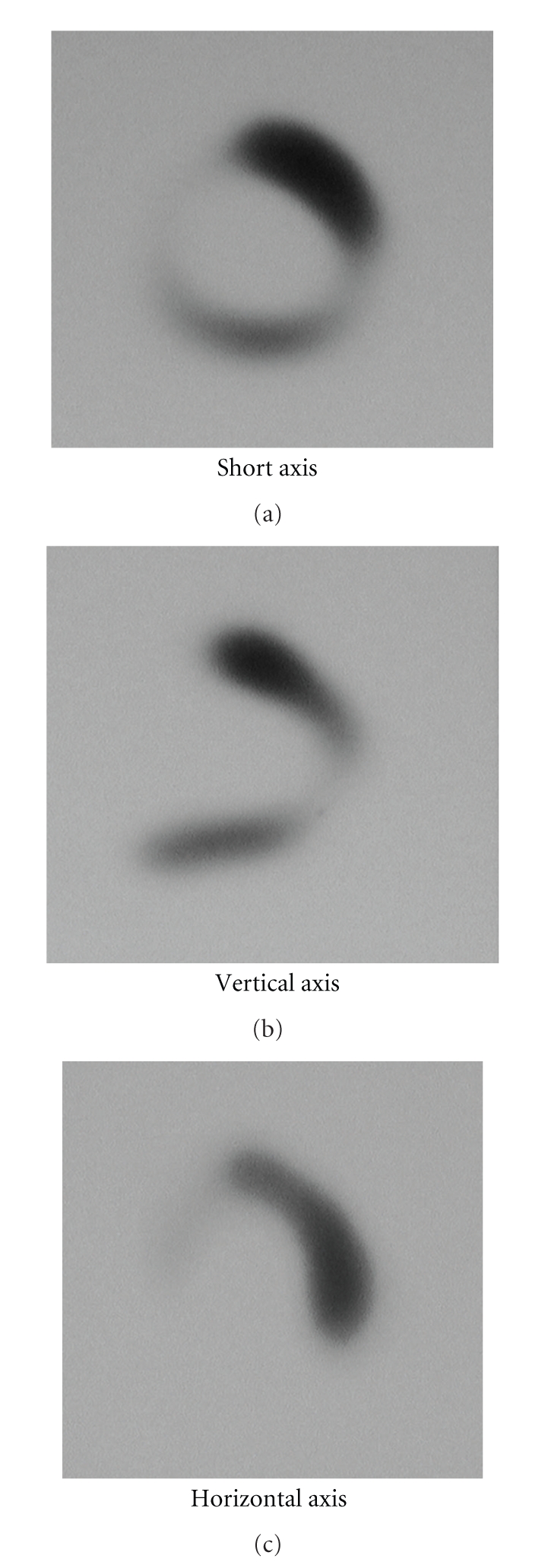
Myocardial scintigraphy with ^99m^Tc-tetrofosmin shows perfusion defect at the septal wall and hypoperfusion at the posterior wall and apex.

Myocardial scintigraphy using ^99m^Tc-tetrofosmin showed a perfusion defect at the ventricular septum and hypoperfusion at the posterior wall and apex, whereas ^67^gallium scintigraphy showed no myocardial uptake. Myocardial biopsy of the RV septum histologically showed epithelioid cell granuloma with infiltration of fibrous cells into the area of lost myocytes (Figure 2), which confirmed myocardial sarcoidosis.

General edema disappeared with furosemide, spironolactone and losartan, while exertional dyspnea and cardiomegaly were sustained. Thus, beta-blocker therapy with carvedilol was started with pimobendan and the dose was step wisely increased to 10 mg/day. When the patient was discharged, exertional dyspnea disappeared and cardiac function was improved to NYHA class II. One year later, echocardiography showed an increment in LVEF to 34% and a shortening of LV end-diastolic dimension to 53 mm, but no change in RV dimension. Right-sided heart catheterization showed pressure reductions in mean pulmonary capillary wedge pressure to 15 mm Hg, RV end-diastolic pressure to 15 mm Hg, and mean right atrial pressure to 17 mm Hg. Three years later, her cardiac function was further improved to NYHA class I. Echocardiography showed further restored LVEF to 54% and LV end-diastolic dimension to 45 mm with moderate tricuspid regurgitation. Throughout the clinical course until then, corticosteroid therapy was not conducted.

## 3. Discussion

The prior history of lung sarcoidosis, the atrioventricular block and the localized myocardial perfusion defect and histologically identified epithelioid cell granuloma have confirmed a diagnosis of cardiac sarcoidosis in this case. Clinical manifestations of cardiac sarcoidosis generally consist of cardiac arrhythmia, electrical conduction disturbance, and congestive heart failure, which occur in less than 5 percent of the patients with sarcoidosis [2–4]. Congestive heart failure mimicking dilated cardiomyopathy in cardiac sarcoidosis has been reported as the consequence of diffuse myocardial fibrosis in cardiac sarcoidosis [5–7]. The present case was characterized by severe biventricular heart failure that subclinically progressed during 14 years after the diagnosis of lung sarcoidosis. The ventricular septum showing the myocardial perfusion defect coincided with the predisposing site in cardiac sarcoidosis [7] and could account for the ECG abnormality. As shown in our case, anteroseptal and apical walls of the LV have been reported as preferentially affected cites in cardiac sarcoidosis [2].

We initially considered that RV enlargement and dysfunction were secondary to LV dysfunction and tricuspid regurgitation. After the conventional medical therapy for heart failure, LV dimension and function were significantly improved, while RV dimension was unchanged despite the reduction in right-sided heart pressure. This suggested that RV enlargement and dysfunction were in part due to RV involvement by sarcoidosis.

## Figures and Tables

**Figure 3:**
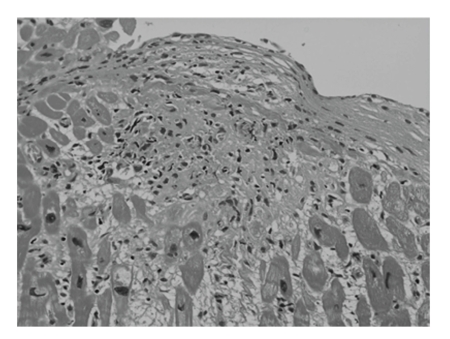
Hematoxylin-Eosin staining of the myocardium from ventricular septum discloses epithelioid cell granuloma.
